# Circular RNA in liver cancer research: biogenesis, functions, and roles

**DOI:** 10.3389/fonc.2025.1523061

**Published:** 2025-03-28

**Authors:** Jiayi Wang, Congcong Zhang, Yinghui Zhang, Jiaojiao Guo, Chenyu Xie, Yulu Liu, Lidian Chen, Liangliang Ma

**Affiliations:** ^1^ The First Affiliated Hospital of Henan University of Chinese Medicine, Zhengzhou, Henan, China; ^2^ School of Rehabilitation Medicine, Henan University of Traditional Chinese Medicine, Zhengzhou, Henan, China

**Keywords:** liver cancer, circRNA, diagnostic biomarkers, therapeutic targets, cancer

## Abstract

Liver cancer, characterized by its insidious nature, aggressive invasiveness, and propensity for metastasis, has witnessed a sustained increase in both incidence and mortality rates in recent years, underscoring the urgent need for innovative diagnostic and therapeutic approaches. Emerging research indicates that CircRNAs (circular RNAs) are abundantly and stably present within cells, with their expression levels closely associated with the progression of various malignancies, including hepatocellular carcinoma. In the context of liver cancer progression, circRNAs exhibit promising potential as highly sensitive diagnostic biomarkers, offering novel avenues for early detection, and also function as pivotal regulatory factors within the carcinogenic process. This study endeavors to elucidate the biogenesis, functional roles, and underlying mechanisms of circRNAs in hepatocellular carcinoma, thereby providing a fresh perspective on the pathogenesis of liver cancer and laying a robust foundation for the development of more precise and effective early diagnostic tools and therapeutic strategies.

## 1 Introduction

Liver cancer ranks among the most common malignant tumors worldwide (1–4). Although various treatment methods exist, their therapeutic effects differ, and they each have specific side effects and limitations (5, 6). Despite therapeutic interventions, the rates of recurrence and metastasis remain elevated, resulting in a poor five-year survival rate (7–9). Additionally,liver cancer exhibits significant tumor heterogeneity and lacks early diagnostic methods, resulting in poor overall survival rates for patients with liver cancer (10–14). Consequently, it is imperative to identify novel therapeutic targets and biomarkers for effective early detection and personalized treatment strategies aimed at enhancing both survival outcomes and quality of life for individuals diagnosed with liver cancer.

Non-coding RNAs (ncRNAs) are integral to cell biology and physiological processes, playing pivotal roles in the pathogenesis of various diseases (15). Among these, circular RNAs (circRNAs), a distinct subclass of ncRNAs found in eukaryotes, have garnered significant attention due to their strong association with the progression of numerous diseases, particularly tumors (16–20). In recent years, a growing body of research has revealed that the abnormal expression of circRNA is intimately associated with the onset and progression of liver cancer. This alteration in expression patterns could have profound implications for the advancement of liver cancer and present novel, promising targets for its diagnosis and prognostic assessment.

This class of endogenous ncRNAs regulates gene expression in various biological organisms; circRNAs are covalently closed-loop RNA transcripts without polyadenylated tails or 5′–3′ polarity (21, 22). This configuration makes circRNA endogenous, abundant in cells (23), presenting higher stability than its linear counterpart and preventing nucleic acid exonuclease-mediated degradation (24). CircRNA exhibits specific expression patterns in tissues and organs, demonstrating significant potential as diagnostic, prognostic, and predictive biomarkers (25–29). With the rapid advancement of high-throughput sequencing technology, scientists have observed that the abundance of certain circRNAs exceeds that of their corresponding linear RNAs by more than tenfold, based on studies of thousands of circRNAs (30). This finding further affirms the crucial role of circRNAs in organisms and provides new insights for studying the mechanisms of circRNAs in liver cancer. Additionally, an extensive sequencing study of more than 2,000 clinical tissue samples and cell lines from over ten types of cancers has shown that circRNAs with stable structures can serve as cancer biomarkers in blood or urine (31).

This manuscript begins with a concise overview of the fundamental characteristics and biological roots of circRNAs. Next, it examines the roles of circRNAs in liver cancer. Lastly, it investigates the potential of circRNAs in diagnosing and treating liver cancer. By systematically studying circRNAs, we aim to offer innovative strategies and approaches for liver cancer diagnosis and treatment, ultimately enhancing patient prognosis and quality of life.

## 2 Historical review of circRNAs

### 2.1 Discovery and early research of circRNAs

The investigation of CircRNAs commenced in 1976 when German researchers, led by Heinz L. Sanger, published findings in the journal PNAS. They confirmed the existence of viroids, which are organisms constituted of single-stranded, covalently closed circular RNA. This pivotal revelation represented humanity’s inaugural approach to understanding circRNA molecules (32). In 1979, Hsu and colleagues utilized electron microscopy to unveil circRNAs in eukaryotic cells (33). By 1993, the identification of circRNAs within human cells became a notable advancement in the field (34).

In the ensuing decades, most circRNAs were regarded as products of mis-splicing or by-products of pre-mRNA processing due to technological limitations and a research focus favoring protein-coding mRNAs (35). The study of circRNAs did not garner much attention. However, with the rapid advancement of high-throughput sequencing (Next-Generation Sequencing, NGS) and bioinformatics in the early 21st century, scientists gradually unveiled the prevalence of circRNAs in eukaryotes and their potential biological functions. After 2010, circRNA research entered a new phase. Researchers began to investigate the functions of circRNAs in depth and discovered their significant roles in gene expression regulation, disease development, and other areas, including serving as protein scaffolds or miR sponges and being translated into peptides (36–38). A circRNA can function as both a miR sponge and a protein template (39–41). In 2012, scientists identified numerous circRNAs in the human body, which prompted increased attention and rapid progress in circRNA research. As research advanced, circRNAs demonstrated close relationships with various biological processes, including development, physiological conditions, and many diseases, such as cancer (**Table 1**).

**Table 1:** Expression of circRNA in various human diseases.

Specific organs	Upregulation	Downregulation
NS	ciRS-7 circBXW7 circHIPK3 circHomer1	circDYM circDLGAP4
BRC	circPLK1 circSKA3 circDENND4C circCDYL	circITCH circCcnb1 circFoxo3
CVD	circLrp6 ciRS-7 circHIPK3 circFoxo3 circNfix circCACNA1D circALPK2 circSPHKAP	circAmotl1 circCDYL1 circANRIL circFndc3b
SC	circPVT1 ciRS-7	circITCH circHIPK3
LC/LF	circTP63 circHIPK3	circFoxo3
DM	circHIPK3 circZNF532 circCIRPB circCAMSAp1	/
HCC	circCDYL circZKSCAN1 ciRS-7	circITCH circMTO1
SMD	circHIPK3 circCDYL circZNF609 circ4099	circGRB10 circAmotl1
CRC	circPVT1 cirRS-7	circITCH circHIPK3
OVC	circHIPK3 circITCH circLARP4	/
BC/PC	circFoxo3 circCDYL	circITCH
KD	circZNF609 circAKT3 circNRIP	/

Thus, the utilization of circRNA in the realms of disease diagnosis, treatment, and prognosis evaluation holds significant value.

## 3 Biological origin of circular RNAs

### 3.1 Biosynthesis of circular RNAs

The biosynthesis of circRNAs is a complex and intricate mechanism centered on the reverse splicing of precursor mRNAs (pre-mRNAs). In this mechanism, the 5’ splice site located downstream of the intron connects with the 3’ splice site positioned upstream in reverse order. This results in the formation of a circular RNA structure, linked by a 3’,5′-phosphodiester bond between the reverse spliced exons (42). CircRNAs are typically derived from 1-5 exons and localize predominantly in the cytoplasm; however, nuclear-localized intron-containing circRNAs (which originate from distinct genomic loci) exhibit unique regulatory functions (43, 44). Based on their sequence types, circRNAs classify into three distinct categories: exonic circRNAs (EcRNAs), intronic circRNAs (CiRNAs), and exon-intronic circRNAs (EIcRNAs) (45–47).

The biosynthesis of circRNAs is a complex and intricate mechanism centered on the reverse splicing of precursor mRNAs (pre-mRNAs). Four primary mechanisms have been elucidated, each involving distinct molecular interactions and regulatory factors:

Mechanism I: “RNA-binding protein (RBPs)-driven cyclization.”

RBPs facilitate the interaction between upstream and downstream introns, promoting circRNA formation. For example, the RBP hnRNPA1 binds to flanking introns of SAMD4 and HIPK3 pre-mRNAs, facilitating Step 1: RBP binding → Step 2: Reverse splicing → Step 3: circRNA maturation (42, 48). This mechanism is particularly relevant in epithelial-mesenchymal transition (EMT) processes.

Mechanism II: “Intron pairing-driven cyclization.”

Reverse complementary sequences in flanking introns form secondary structures, enabling cyclization. The CDR1as locus exemplifies this mechanism, where 70 conserved Alu elements mediate circularization and miR-7 sequestration (49). Introns may subsequently be removed or retained, leading to the formation of ecircRNAs or EIciRNAs (50).

Mechanism III: “Lariat-driven cyclization.”

During canonical splicing, exon skipping generates a lariat intermediate containing exons and introns. This process can generate a lasso structure containing an exon and an intron. If the introns are subsequently removed, ecircRNA or EIciRNA forms (51).For instance, the SAMD4 gene produces circRNAs via this mechanism, with the lariat structure stabilized by U2AF65 and SF1 splicing factors (50).

Mechanism IV: “lasso intron-driven cyclization.”

CiRNAs (circular intronic RNAs) evade debranching enzyme degradation through specific motifs. The ci-ankrd52 locus retains a 7-nt GU-rich element near the 5′ splice site and an 11-nt C-rich element at the branchpoint, forming a stable lariat structure (44). This binding enables circRNAs to evade degradation by debranching enzymes, thereby forming stable ciRNAs (52). Together, these mechanisms highlight the complexity and diversity of circRNA biological origins.

Overall, circRNA biosynthesis constitutes a complex and delicate process involving multiple steps and regulatory factors. With ongoing research, we anticipate gaining a more comprehensive understanding of circRNA biosynthesis mechanisms and uncovering its significant functions in biology and medicine.

### 3.2 Functions of circular RNA

#### 3.2.1 Regulation of gene expression

Circular RNAs exert multifaceted roles in modulating gene expression through diverse mechanisms:

##### 3.2.1.1 miRNA sponge activity

CircRNAs act as ceRNAs or miR sponges and are one of their most notable functions (53, 54). miRNAs are a class of short non-coding RNA molecules that inhibit the transcription and translation process of target genes by binding to their mRNAs (55), whereas circRNAs have multiple miRNA-binding sites that compete for binding to miRNAs, thereby regulating miRNA activity and affecting miRNA-regulated target gene expression (56, 57). CDR 1as, for example, carries 63 conserved miR-7 binding sites, which significantly affect the expression of tumor-associated genes by enhancing the stability of miR-7 target mRNAs, which in turn are tightly linked to the process of tumor progression (49, 58, 59). In triple-negative breast cancer research, circCD 44 has been identified as a sponge for miR-502-5p, effectively sequestering and inhibiting its activity, thereby contributing to the initiation and progression of tumorigenesis (60). Similarly, the overexpression of circLRP 6 has also been empirically validated to accelerate the pathological progression of atherosclerosis by absorbing miR-145, further emphasizing the regulatory role of circRNAs in modulating disease processes (61).

##### 3.2.1.2 Interaction with RNA-binding proteins

RNA-binding proteins (RBPs) serve as critical regulators of RNA metabolism and play significant roles in various RNA processes (62). Recent investigations have revealed that circRNAs can bind to RBPs, thereby co-regulating gene expression (42, 63–65). For instance, both two circRNAs—circBACH1 and circZKSCAN1—have been implicated in liver cancer progression; they modulate the expression of oncogenic genes and associated signaling pathways through interactions with distinct RBPs (66–70). Additionally, specific circRNAs can modify the cellular localization of RBPs, enabling these proteins to execute specialized functions in non-canonical contexts, which provides novel mechanistic insights into their regulatory mechanisms (66).

CircRNAs interact with RBPs to modulate RNA transcription and translation processes. These circular RNAs possess unique structures that allow them to specifically bind RBPs and forming stable protein-RNA complexes. Such interactions can modulate key aspects of RBP function, stability, and activity of these proteins, further impacting RNA transcription and translation. Consequently, circRNAs serve as critical regulators of RNA metabolism and global gene expression.

##### 3.2.1.3 Direct transcriptional regulation

Circular RNAs, specifically circSEP3 and circSMARCA5, act as transcriptional regulators. They induce pauses and termination in transcription at specific exons by binding directly to DNA, which alters gene expression patterns (71, 72). In contrast, EIciRNA enhances gene expression through its interaction with U1 small nuclear ribonucleoprotein (46). Additionally, cyclic intronic RNAs (ciRNAs) accumulate at their production sites, boosting gene expression by regulating the activity of RNA polymerase II (73). CircRNAs function as crucial transcriptional regulators, engaging with transcription factors and regulatory elements. This interaction influences the formation and activity of transcription complexes, thereby impacting the transcription levels of targeted genes. Moreover, circRNAs can directly modulate gene expression via chromatin interactions. These multifaceted roles position circRNAs as vital players in cell fate determination, tissue development, and the initiation of diseases.

#### 3.2.2 Protein-related functions

Circular RNAs directly influence protein activity, localization, and translational dynamics through structural and scaffolding mechanisms:

##### 3.2.2.1 Regulation of protein translation

Researchers have identified translatable circRNAs (74, 75), and recent investigations have confirmed their widespread existence (76, 77). Although circRNAs do not directly encode proteins, they play a role in protein translation by interacting with proteins. They function as “molecular adsorbents,” binding proteins to form complexes. This interaction can modify the stability, subcellular localization, and activity of proteins. Such associations may influence protein synthesis, folding, transport, and degradation, thereby regulating the intracellular protein balance and functionality.

##### 3.2.2.2 Scaffolding function for protein complexes

CircRNAs function as scaffolds for protein complexes by interacting with a range of proteins. This scaffolding role enhances protein interactions and signal transduction, thereby modulating intracellular signaling pathways and biological processes. By forming these protein assemblies, circRNAs coordinate the actions of multiple proteins, facilitating intricate cellular regulation. Moreover, circRNAs are instrumental in fostering interactions between two or more proteins. Notable examples include circ-Amotl1 and circ-Foxo3, which act as protein scaffolds to help align enzymes with their substrates. For instance, circ-Amotl1 has been recognized as a scaffold that promotes interactions with PDK1 and AKT1 (78), facilitating their movement into the nucleus, a vital process for cell growth and survival. Furthermore, a study by Du et al. underscored the role of circ-Foxo3 as a scaffold for a variety of proteins, demonstrating its ability to bind with p53 and the E3 ubiquitin-protein ligase Mdm2 (79). This interaction enhances Mdm2-driven ubiquitination and the subsequent degradation of p53.

**Figure 1:**
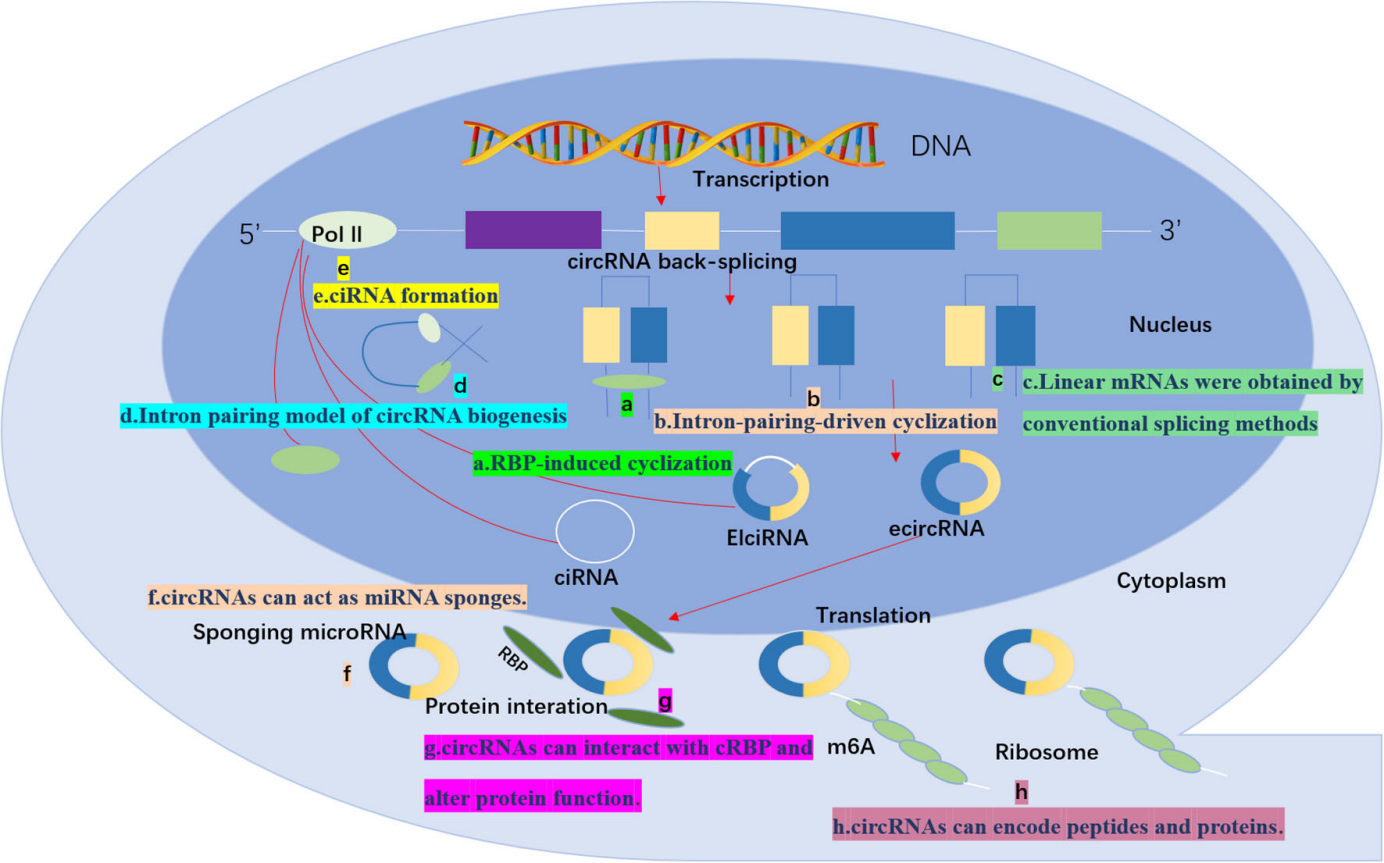
CircRNA biogenesis and function: Typical reverse splicing produces three types of circRNAs: ciRNAs, EIciRNAs, and ecircRNAs. function **(a)** RBP-induced cyclization: the RBP facilitates a reverse splicing event in which introns are removed to form circRNAs. **(b)** Intron-pairing-driven cyclization; reverse complementary sequences (Alu sequences) direct the insertion and by removing or retaining the introns generates EIciRNA or ecircRNA. **(c)** Linear mRNAs were obtained by conventional splicing methods **(d)** Intron pairing model of circRNA biogenesis: a 3’ splice donor site in an exon binds to a 5’ splice acceptor site in an upstream intron to form circRNAs or ecircRNAs by removing introns between exons. ecircRNA is formed by removing the intron between the exons. **(e)** ciRNA formation; ciRNA is derived from the lactinuclear intron. **(f)** circRNAs can act as miRNA sponges. **(g)** circRNAs can interact with cRBP and alter protein function. **(h)** circRNAs can encode peptides and proteins. This image was made using Microsoft PowerPoint.

In conclusion, circRNAs are vital in functions such as miRNA sponging, RNA-binding protein interactions, gene transcription regulation, protein translation, and acting as binding protein scaffolds (**Figure 1**). These roles position circRNAs as key players in managing intracellular gene expression and protein functionality, crucial for insights into cell biology and disease mechanisms. As research progresses, we anticipate uncovering additional functions and regulatory pathways of circRNAs, offering novel perspectives and approaches for disease diagnosis and treatment

## 4 Relationship between circular RNA and liver diseases

### 4.1 CircRNA in non-alcoholic steatohepatitis

Non-alcoholic fatty liver disease (NAFLD) is characterized by the abnormal buildup of fat in the liver. If left untreated, NAFLD can advance to nonalcoholic steatohepatitis (NASH), which represents a more severe state (80). The frequency of both NAFLD and NASH has notably increased due to shifts in lifestyle and contemporary dietary practices. These liver conditions are closely associated with cirrhosis and liver cancer (81). Day and James hypothesized that steatosis serves as a possible second insult leading to NASH (82). Recent research has unveiled abnormal miRNA expression, excessive triglyceride build-up, and lipid peroxidation dysregulation as key contributors to hepatic steatosis. Importantly, miR-34a is crucial in this context; circRNA_0046367 finely tunes its function (83). The expression of circRNA_0046367 declines significantly in the context of hepatic steatosis. Restoring its levels can effectively diminish lipid peroxidation, reduce apoptosis, and ameliorate mitochondrial dysfunction. This recovery lifts the inhibitory action of miR-34a on peroxisome proliferator-activated receptor α (PPARα), subsequently enhancing the expression of genes involved in lipid metabolism (84). Additionally, studies indicate that circ_0046366 is another circRNA linked to NAFLD pathogenesis, functioning as an antagonist of miR-34a. The absence of this circRNA prominently contributes to hepatocellular steatosis induced by a high-fat diet. Fortunately, reinstating circ_0046366 expression effectively hampers the steatosis progression, deactivates miR-34a, and spurs the transcriptional activation of lipid metabolism-related genes. This process restores compromised PPARα activity, representing a novel strategic approach for NAFLD treatment. While a direct link between circRNAs and liver disease metabolism has yet to be established, the miRNAs discovered in this study, which interact with differentially expressed circRNAs, undeniably present valuable insights for future investigations into these non-coding RNAs within the NAFLD/NASH pathology.

### 4.2 CircRNA in liver cirrhosis

Liver fibrosis is an end-stage liver disease that develops from chronic liver injury and can lead to cirrhosis with a poor prognosis. Studies have shown that one of the key mechanisms of liver fibrosis is the activation of hepatic stellate cells (HSCs), which transform from a resting state to myofibroblasts and promote fiber formation (85). It was shown that 179 circRNAs were found to be up-regulated and 630 down-regulated in radiation-induced activation of HSCs, indicating significant changes in circRNA expression, and in particular, silencing of hsa_circ_0071410 increased miR-9-5p expression and inhibited HSCs activation (86). These data suggest that circRNAs may play an important role in NAFLD/NASH-associated hepatic fibrosis, but the specific mechanisms need to be thoroughly investigated.

### 4.3 CircRNA in liver cancer

#### 4.3.1 Expression characteristics and changing law of circular RNA in liver cancer

CircRNA is a closed ring RNA molecule that exhibits greater stability and resistance to RNase degradation compared to linear RNA. In liver cancer tissues and cells, circRNA expression patterns often show tissue specificity and tumor relevance. Studies reveal that the expression levels of numerous circRNAs in liver cancer differ significantly from those in normal liver tissues, closely linking them to the clinicopathologic features of liver cancer. While the specific functions and mechanisms of circRNAs in liver cancer require further exploration, many reports confirm their pivotal roles in this disease (87–97).

In terms of cell proliferation and carcinogenesis, CircRNAs affect cell proliferation and tumorigenesis in liver cancer progression. Some CircRNAs express highly at the early stage of liver cancer and accelerate the malignant proliferation of tumor cells by regulating specific genes and signaling pathways. One study summarized the following signaling pathways through which CircRNAs regulate tumor proliferation and metastasis (98).

The specifics are as follows:

1. The Wnt/β-catenin pathway is a signaling pathway closely related to tumor progression. In liver cancer, circMTO1 activates the Wnt/β-catenin signaling pathway by regulating the miR-541-5p/ZIC1 axis, which in turn inhibits tumor proliferation. In contrast, hsa_circRNA_104348 activated the pathway through the miR-187-3p/RTKN2 axis and promoted cell proliferation. CircRNA-SORE acted as a sponge for miR-103a-2-5p and miR-660-3p, and similarly competed to activate the Wnt/β-catenin signaling pathway. In contrast, circZFR further enhances the activation of the Wnt/β-catenin signaling pathway and promotes proliferation by regulating miR-3619-5p/CTNNB1 (99–102).
2. The nuclear factor-κB (NF-κB) signaling pathway is also closely related to proliferation. circZFR promotes liver cancer development by inhibiting the STAT3/NF-κB pathway (103). CircLIFR, on the other hand, promotes cell proliferation by interacting with TBK1, a serine/threonine kinase that regulates the NF-κB pathway (104); In contrast, circCORO1C further promotes liver cancer proliferation and metastasis by upregulating NF-κB pathway-induced PD-L1 expression (105).
3. The PI3K/AKT signaling pathway plays a critical role in regulating multiple cellular functions. In liver cancer, circCDYL, circCDK13, and circEPHB4 regulate the proliferative capacity of liver cancer cells through the PI3K/AKT signaling pathway (89, 106, 107).
4. In the context of liver cancer, the mitogen-activated protein kinase (MAPK) signaling pathway, as a series of highly conserved enzymatic cascades, plays a crucial role in physiological activities such as cell proliferation, differentiation, and apoptosis. CircDHPR targets the RASGEF1B/RAS/MAPK signaling pathway by interacting with miR-3194-5p, which in turn promotes tumor growth and metastasis (108). In addition, circASAP1 acts as a sponge for miR-326 and miR-532-5p and regulates MAPK1 expression, thereby enhancing cell proliferation and invasion (88). In contrast, circSETD3 exerts an inhibitory effect on tumor growth by targeting the miR-421/MAPK14 signaling pathway (109).

Many circRNAs also regulate pathways that impact liver cancer progression. By influencing tumor-related signaling pathways, circRNAs ultimately affect tumor progression, making this a promising research direction. circIPO11, for instance, is a conserved circRNA that shows high expression in liver cancer tumors and hepatic CSCs, and plays a role in maintaining hepatic CSC self-renewal. It promotes liver CSC self-renewal and advances liver cancer proliferation through the activation of the Hedgehog signaling pathway. Antisense oligonucleotides (ASOs) that target circIPO11 exhibit synergistic antitumor effects when combined with the TOP1 inhibitor camptothecin (CPT) (110). The nuclear circRNA circASH2 is absent in liver cancer tissues and suppresses liver cancer metastasis by modifying the structure of the tumor cytoskeleton. Proto-myosin 4 (TPM4), the primary target of circASH2, is repressed post-transcriptionally. CircASH2 enhances the liquid-liquid phase separation of nuclear Y box binding protein 1 (YBX1), thus promoting the decay of TPM4 transcripts, highlighting the role of tumor suppressor circRNAs and their intricate regulatory mechanisms in liver cancer progression (111).

Second, circRNAs play a key role in cell movement and infiltration during liver cancer progression. Cellular transformation is a complex process that enables cancer cells to gain enhanced movement and infiltration capabilities. Certain circRNAs promote cancer cell migration and invasion by regulating associated genes and signaling pathways. For example, circARFGEF2 significantly accelerates the metastasis of liver cancer in the liver and lung by regulating specific signaling pathways (96).

In addition, circRNAs contribute to the regulation of immune system function and influence liver cancer development. Immune dysfunction closely relates to liver cancer progression, and circRNAs can regulate the activity and function of immune cells, affecting the immune evasion of cancer cells. For example, circUHRF1 inhibits the immune function of NK cells by upregulating TIM-3 expression in NK cells, thus promoting immune evasion by cancer cells (112).

**Figure 2:**
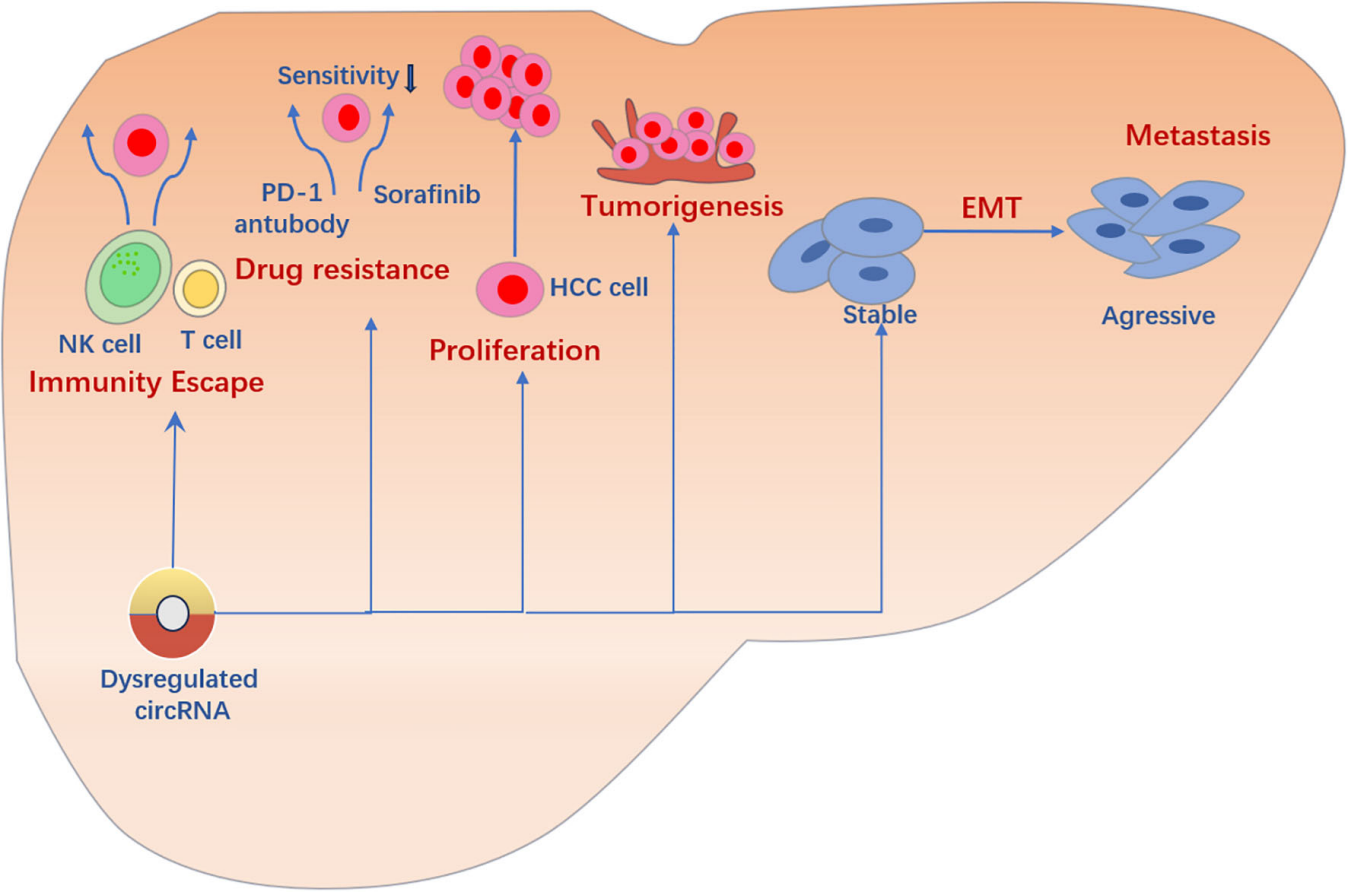
Multiple roles of CircRNA in liver cancer:circRNA plays multiple roles in liver cancer. It regulates cell proliferation and tumor initiation, participates in the EMT process, and promotes invasion and metastasis of liver cancer cells. In addition, circRNA affects the progression and prognosis of liver cancer by regulating the immune system. Notably, some circRNAs may induce and maintain drug resistance in liver cancer treatment, challenging therapeutic strategies.This image was made using Microsoft PowerPoint.

Finally, circRNAs may play an important role in drug response. Although researchers have not fully understood the specific mechanisms of circRNAs in drug response, evidence shows that certain circRNAs may regulate drug effects and contribute to drug resistance. Studies in this area will deepen our understanding of the mechanisms of drug response in liver cancer and provide new insights for future therapeutic strategies.In summary, the pathogenesis of liver cancer is closely related to the function of circRNAs (**Figure 2**).

#### 4.3.2 Diagnostic value and prognostic significance of circular RNA in liver cancer

Due to the tissue specificity and stability of circRNA expression, many studies now explore its potential in the early diagnosis of liver cancer. Compared with traditional serum markers like AFP, circRNAs demonstrate higher specificity and sensitivity. Researchers found that changes in the expression levels of specific circRNAs in liver cancer tissues and blood samples effectively differentiate liver cancer patients from healthy controls, providing better diagnostic value. For instance, Conn et al. (48) discovered that circRNA_circFOXP1 promoted the infiltration and migration of liver cancer by regulating cell-cell interactions within the liver cancer microenvironment. Its high expression in liver cancer tissues closely linked to deep tumor infiltration and poor prognosis. Hence, circRNA can serve as a novel diagnostic marker for liver cancer, potentially improving the accuracy and efficiency of early diagnosis.

**Figure 3:**
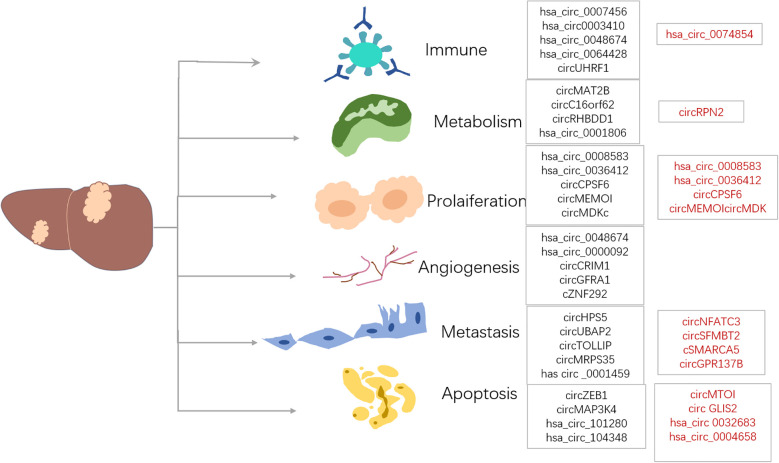
CircRNAs are closely related to liver cancer, and they are associated with processes such as proliferation, metabolism, angiogenesis, metastasis, immune regulation and apoptosis. In these processes, CircRNAs may either act as oncogenes (marked in black font), promoting tumor development, or play the role of suppressor genes (marked in red font), inhibiting the tumor process, demonstrating their complex and diverse functions. This image was made using Microsoft PowerPoint.

Moreover, numerous studies on liver cancer patients reveal that hsa_circ_0000798 (113), hsa_circ_0027089 (114), and hsa_circ_0058124 (115) are up-regulated in liver cancer tissues. In contrast, some circRNAs down-regulate, including hsa_circSMARCA5 (116), hsa_circ_0068669 (117), hsa_circ_0028502 (118), and hsa_circ_0076251 (118). These circRNAs are regarded as potential biomarkers for liver cancer. Additionally, some circRNAs play roles in several hallmarks of cancer, including cell death and survival, invasion, metastasis, and angiogenesis. Various validation tests, such as northern blotting, spot blotting, RNA-seq, and circRNA-specific microarrays, show significant dysregulation of many key circRNAs in liver cancer cells, tissues, blood, and exosomes (**Figure 3**).

#### 4.3.3 Potential and feasibility of circular RNA as a therapeutic target in liver cancer

In liver cancer, a variety of specific circRNAs show aberrant expression, and these molecules occupy a central role in the progression of liver cancer, and thus are considered as potential diagnostic indicators and therapeutic targets for liver cancer (119–121). circRNAs can be found in tissues (122), exosomes (123–125), plasma (31), serum (126), cerebrospinal fluid (127), urine (31), saliva (128), and other biological samples were detected. Due to the structural property of reverse splicing of circRNAs, they are able to avoid recognition by nucleic acid exonucleases and therefore have a longer half-life compared to linear RNAs. This stability makes circRNAs ideal candidates for cancer diagnosis, monitoring and treatment (129–131).

Numerous studies have revealed the potential application of circRNAs as cancer biomarkers (132–137). However, in-depth evaluation of their diagnostic accuracy is lacking. For instance, high expression of circASAP 1 strongly associates with poor prognosis, lower survival, and higher recurrence rates in liver cancer (88). Additionally, the expression levels of hsa_circ_0001955 (138) and circ_104075 (139) increase in the tissues, serum, and plasma of liver cancer patients. Notably, the increased expression of circ_104075 is specific to liver cancer, as similar increases do not occur in other liver diseases or tumors (139). Serum levels of both circRNAs significantly reduce after surgical resection of the tumor tissue, suggesting that hsa_circ_0001955 and circ_104075 may serve as biomarkers for assessing the efficacy of surgical treatment and the risk of tumor recurrence. Moreover, DHX 9 expression significantly upregulates in liver cancer, inhibiting the production of cSMARCA 5 (hsa_circ_0001445) by binding and inhibiting the pairing of flanking reverse-complementary sequences. This, in turn, hampers the production of cSMARCA 5 (hsa_circ_0001445) (140). As a tumor suppressor, cSMARCA 5 inhibits liver cancer growth through the cSMARCA 5/miR-17-3p/miR-181b-5p/TIMP3 pathway. A decrease in cSMARCA 5 in liver cancer tissues correlates with increased tumor growth and metastasis, making it an independent prognostic indicator for patients post-tumor resection (87).

### 4.4 CircRNA in exosomes and liver cancer

In studies exploring the relationship between circRNAs in exosomes and liver cancer, we observed a significant overexpression phenomenon of circRNA Cdr1as in exosomes released by liver cancer cells, a phenomenon that promotes the proliferation and migration ability of neighboring normal cells. circ-ZEB 1 and circ-AFAP 1 are associated with stemness and prognosis of liver cancer poorly and can regulate the epithelial-mesenchymal transition (EMT) process. Our novel exosome-derived circRNAs play crucial roles as key components of various intercellular crosstalk and communication systems in malignant transmission. This finding provides valuable support for the use of plasma exosomal circRNA as a clinical prognostic indicator for liver cancer patients (141). zhang et al. found that adipocyte-derived circ-DB promoted tumor growth and affected DNA damage repair through miR-34a and USP7 regulation (142), and Lai et al. indicated that circFBLIM1 was found in serum exosomes and liver cancer cells are highly expressed, and its inhibition limits glycolysis and liver cancer progression (143), and Huang et al. showed that circRNA-100338 in MHCC97H exosomes promotes liver cancer cell invasion and metastasis (144). The selective sorting of circRNAs into exosomes involves interactions with RNA-binding proteins (RBPs) such as GW182 and ELAVL1, which recognize specific sequence motifs (e.g., GGAG/CCCU) in circRNA loops (141). The nSMase2/sphingomyelinase pathway regulates ceramide production, promoting exosome biogenesis and circRNA loading (142). The exosome circANTXR 1 correlates with clinical characteristics (TNM stage and tumor size) and poor prognosis of liver cancer patients, and the circANTXR 1/miR-532- 5 p/XRCC 5 axis-mediated inhibition of liver cancer progression may be an effective strategy for treatment (145). For the association between high expression of exosomal circAKT 3 and poor prognosis, circAKT 3 could be used as a surveillance biomarker for early detection of recurrence (146). sun et al. identified three circRNAs (hsa_circ_0004001, hsa_circ_0004123, hsa_circ_0075792) as highly sensitive and specific biomarkers for liver cancer diagnosis (147), and Chen et al. found that circ-0051443 was delivered to liver cancer cells via exosomes, regulated miR-331-3p and BAK1, and inhibited the malignant behavior of liver cancer (148). The above studies not only deepened our understanding of the mechanism of circRNA action in liver cancer, but also suggested the important role of exosomes as circRNA delivery mediators in the regulation of tumor microenvironment. These findings provide new perspectives and potential targets for the development of circRNA-based therapeutic strategies for liver cancer, and are expected to provide new ideas and approaches for clinical intervention. A growing number of studies have indicated that some serum exosomal circRNAs also have potential as biomarkers for prognosis and tumor monitoring. As shown in the **Table 2**.

**Table 2:** Overview of dysregulated serum exosomal circRNA in liver cancer.

CircRNA Name	Year	Expression in liver cancer	Regulatory Axis	Function	Ref
ciro-DB	2018	Upregulated	miR34N USP7	Promoting tumor growth and reducing DNA damage	(142)
cirePTGRI	2019	Upregulated	miR449x/MET	Increasing migratory and invasion and metastasis. potential prognosis biomarker	(150)
Cdrlas	2019	Upregulated	miR-1270/AFP	Promoting prolierative and migratory	(151)
hsa_circ_0051443	2020	Downregulated	miR-331-3p/BAKI	Suppressing liver cancer progression, promoting cell apoptosis and arresting the cell cycle, diagnosis biomarker of liver cancer	(148)
circRNA-I00338	2020	Upregulated	Not provide	Enhancing invasiveness and angiogenesis.promoting metaseasis, as a risk indicator of pulmonary metastasis and poor survival.	(144)
circFBLIMI	2020	Upregulated	miR-33B/LRP6	Promoting liver cancer progression and glycolysis	(143)
circRNA·SORE	2020	Upregulated	YBXI	Mediating sorafenib resistance in liver cancer	(152)
hsa_circRNA_104348	2020	Upregulated	miR-187-3p	promoting proliferation, suppressed apoptosis of liver cancer cells	(100)
circUHRFI	2020	Upregulated	miR-449c.5p/TIM-3	Contributing to immunosuppression by inducing NK cell dysfunction in liver cancer, casuing resistance to anti-PD1 immunotherapy	(112)
circTMEM45A	2020	Upregulated	miR-665/IGF2	Promoting cell mobility *in vitro*, as well as *in vivo* tumorigenesis,acting as a diagnosis biomarker	(153)
circAKT3	2020	Upregulated	Not provide	Associating with a higher risk of liver cancer recurrence and mortality	(146)
circANTXRI	2021	Upregulated	miR-532-5p/XRCC5	Promoting the proliferation, migration and invasion of liver cancer cells	(145)
circGPR137B	2022	Downregulated	miR-4739	inhibiting liver cancer tumorigenesis and metastasis through the circGPR137B/miR-4739/FTO feedback loop.	(154)
circRPN2	2022	Downregulated	miR-183- 5 p/FOXO 1	Inhibiting Aerobic Glycolysis and Metastasis in liver cancer	(155)
hsa_circ_0002003	2023	Upregulated	miR-1343- 3 p/STMN 1	promoting liver cancer progression	(156)
circPIAS 1	2024	Upregulated	circPIAS 1	enhancing iron storage in liver cancer cells and conferring resistance to ferroptosis	(157)

## 5 Conclusion and outlook

Emerging evidence from liver cancer research underscores the pivotal role of circRNAs in modulating gene expression networks and cellular signaling pathways. These regulatory mechanisms not only elucidate the pathophysiological basis of hepatocellular carcinoma but also provide actionable insights for developing circRNA-based therapeutics, including innovative biomarkers and precision-targeted anticancer interventions (149).It has gradually attracted extensive attention from researchers. Despite the remarkable progress in circRNA research related to liver cancer, several challenges still remain.

### 5.1 Methodological limitations and technological advancements

While single-cell RNA-seq and spatial transcriptomics have revolutionized our understanding of circRNA heterogeneity, their application remains constrained by:

Current research on circRNA function primarily relies on bioinformatics predictions and *in vitro* experiments but lacks *in vivo* validation. Meanwhile, researchers have not yet completely clarified the target genes and mechanisms of action of circRNAs, and the specific interaction network between circRNAs and liver cancer requires further elucidation. The multiple cell types within the liver, such as hepatocytes, hepatic stellate cells, blast cells, and immune cells, also complicate circRNA studies in liver disease. Future studies should aim to expand the functional spectrum of circRNAs, thoroughly resolve their regulatory networks, and utilize advanced technological tools like single-cell sequencing and functional genomics to uncover the mechanisms of their roles in liver cancer development and progression. More in-depth studies are necessary.

Future directions:

1.Develop integrative pipelines combining single-cell circRNA profiling with patient-derived organoids to bridge preclinical and clinical data gaps.
2. Leverage AI-driven models (e.g., Transformer-based architectures) to predict circRNA-protein interaction networks.

### 5.2 Limitations of sample size and dataset

Existing studies mainly rely on small sample sizes and heterogeneous datasets, which restrict the broad application and dissemination of the results. Large-scale, standardized clinical samples and datasets will enhance the reliability and accuracy of the studies.

Future directions:

1. Large-Scale Cohort Studies: Initiatives like the Liver Cancer Precision Medicine Consortium provide unparalleled opportunities to analyze circRNA signatures across diverse ethnicities and disease stages.
2. Standardized Data Repositories: Expanding public databases with uniformly processed RNA-seq and clinical metadata will accelerate meta-analytic discoveries.

### 5.3 Challenges of clinical translation

Although circRNA has shown great potential in laboratory research, its translation into clinical applications still faces many challenges, such as drug delivery, stability, and specificity.

Circular RNA, as a novel non-coding RNA, holds significant theoretical and applied value in the study of liver cancer. Future studies can further explore the potential of circRNA for early diagnosis and prognostic assessment of liver cancer, develop new strategies for circRNA-targeted therapy, and utilize circRNA in individualized therapy. Additionally, by combining bioinformatics analysis with clinical data, researchers can establish a multidimensional database of circRNA to facilitate its application as a biomarker. In summary, further in-depth exploration of circRNA in liver cancer research will lead to new breakthroughs and advancements in liver cancer treatment and management.
